# Immunotherapy-induced sialadenitis: sjögren’s syndrome or a new sialadenitis

**DOI:** 10.3389/fimmu.2026.1755419

**Published:** 2026-02-27

**Authors:** Shuyuan Song, Zhentao Lao, Ruotong Yu, Shan Yu, Peiyao Li, Yumeng Yan, Le Yang, Guiqing Liao, Yan Wang, Sien Zhang

**Affiliations:** 1 Hospital of Stomatology, Guanghua School of Stomatology, Sun Yat-sen University, Guangzhou, Guangdong, China; 2 Guangdong Provincial Key Laboratory of Stomatology, Sun Yat-sen University, Guangzhou, Guangdong, China

**Keywords:** chronic sialadenitis, head and neck squamous cell carcinoma (HNSCC), immune checkpoint inhibitors, CD4^+^T cells, Th17/IL-17 axis

## Abstract

**Objective:**

Although immune checkpoint inhibitors (ICIs) have improved survival in head and neck squamous cell carcinoma (HNSCC), associated adverse events, such as sialadenitis, remain poorly characterized. This study aimed to define the clinicopathological features, establish the causal pathogenic mechanism, and validate a therapeutic target for ICI-associated sialadenitis.

**Methods:**

This study integrated three complementary approaches. First, a prospective cohort of 25 HNSCC patients underwent functional assessment of salivary and lacrimal glands before and after ICI therapy. Second, salivary gland tissues from separate cohorts of ICI-treated (n=30) and untreated control (n=30) patients were subjected to comprehensive analysis, including histology, multi-platform immunophenotyping (immunohistochemistry, multiplex immunofluorescence, flow cytometry), and cytokine quantification at both transcript and protein levels. Finally, a preclinical mouse model was established to confirm causality and validate the therapeutic efficacy of IL-17A blockade.

**Results:**

Following ICI treatment, patients showed significantly reduced salivary and lacrimal secretion (*P <* 0.05). Histopathological analysis revealed extensive lymphocytic infiltration, marked periductal fibrosis, and substantial loss of acinar structures. The immune infiltrate was dominated by CD4^+^ T cells, particularly the Th17 subset, with corresponding upregulation of IL-17A both at transcriptional and protein levels. Crucially, we established a mouse model of anti-PD-1-induced sialadenitis and demonstrated that therapeutic blockade of IL-17A restores salivary function.

**Conclusion:**

This study establishes ICI-associated sialadenitis as a distinct pathological entity characterized by CD4^+^T cell-driven inflammation mediated through the Th17/IL-17 axis, which differs from Sjögren syndrome, predominantly involving B cells and from IgG4 related sialadenitis. By demonstrating therapeutic efficacy in a preclinical model, our findings provide the first preclinical validation of the IL-17 axis as an actionable therapeutic target for this condition.

## 1 Introduction

Immune checkpoint inhibitors (ICIs) have revolutionized cancer therapy, particularly in the treatment of head and neck squamous cell carcinoma (HNSCC). Although their therapeutic benefits are substantial, ICIs frequently trigger immune-related adverse events (irAE) affecting multiple organs, including endocrine glands (1–5), exocrine glands (6–8), skin (9), gastrointestinal tract (10), and liver (11, 12). In particular, Cappelli et al. reported that sicca symptoms affecting the eyes and mouth occur in 30.7% of patients receiving ICI therapy, substantially impacting their quality of life (13). Despite their clinical significance, the underlying mechanisms of these complications remain poorly understood, which hinders the development of effective prevention and management strategies.

The salivary glands play an essential role in oral health through their production of saliva, which maintains dental mineral balance, provides mucosal protection, and establishes crucial antimicrobial defenses (14, 15). Although ICI-induced sialadenitis is increasingly recognized, its pathophysiology remains controversial (16, 17). Some researchers classify it as ICI-induced Sjögren’s syndrome (SjS) (18, 19); however, distinct clinical and histological features challenge this classification (20–24) (25, 26). Unlike classic SjS, it does not exhibit a female predominance (27), lacks characteristic autoantibodies (anti-SSA/SSB) (27, 28), and demonstrates predominantly T-cell rather than B-cell-rich infiltrates (16, 29). These differences raise a crucial question of whether ICI-induced sialadenitis is a variant of SjS or should it be recognized as a distinct pathological entity.

To bridge this knowledge gap, we conducted a comprehensive study integrating clinical observation, human tissue analysis, and a preclinical animal model. We first identified a unique CD4^+^ T cell-dominant, Th17-skewed inflammation with elevated IL-17A in patients with HNSCC undergoing ICI therapy. To test the functional significance of this pathway, we generated a mouse model of sialadenitis induced by anti-PD-1 therapy. Critically, therapeutic blockade of IL-17A in this model restored glandular function. Thus, our findings identify ICI-induced sialadenitis as a Th17-driven pathology and highlight the IL-17 axis as a promising druggable target.

## 2 Results

### 2.1 ICI therapy induced severe glandular hypofunction and sicca-like symptoms

**Figure 1:**
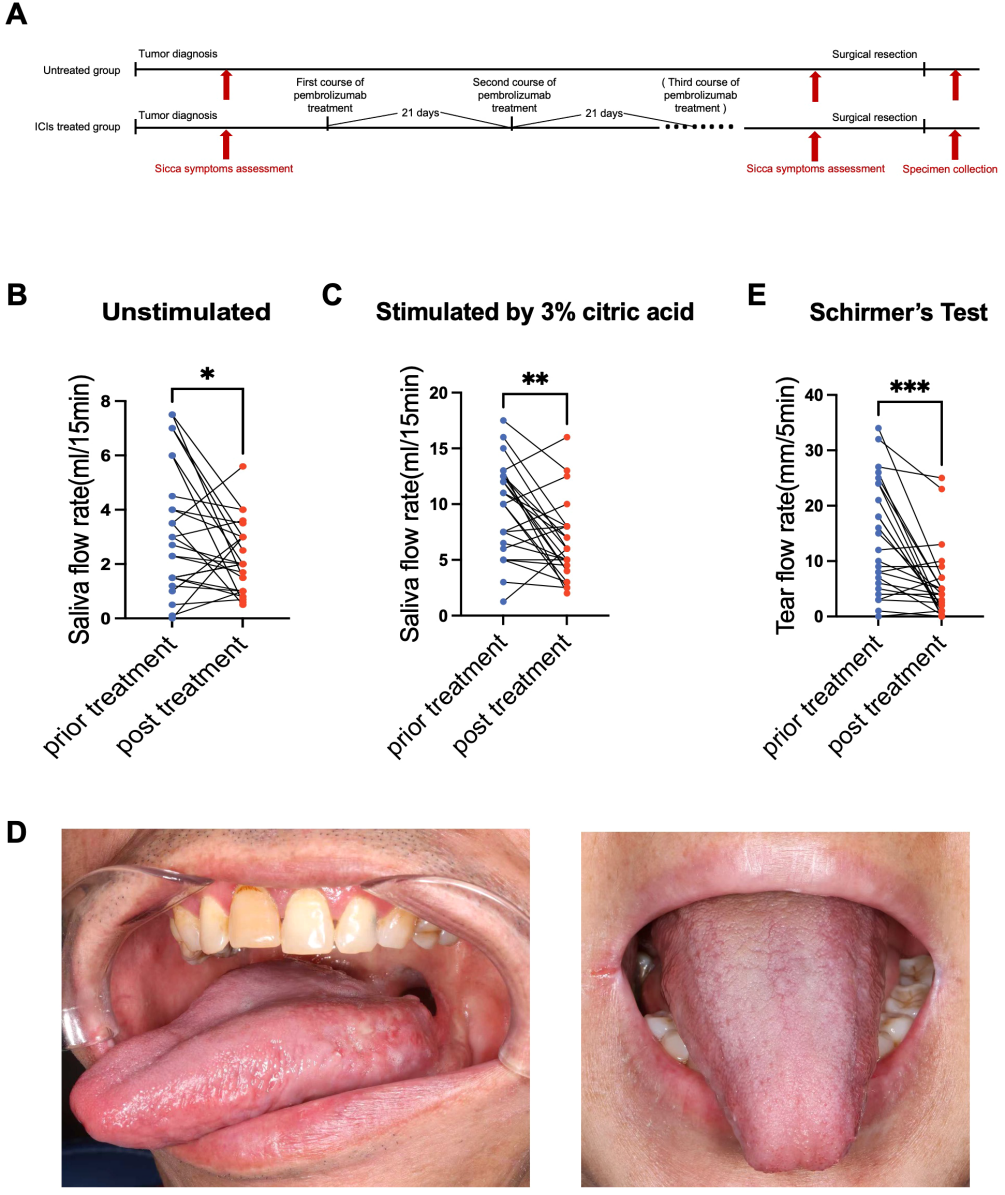
ICI therapy induces severe glandular hypofunction and sicca-like symptoms. **(A)** Flowchart of patient enrollment, treatment, clinical testing, and specimen collection. **(B)** Unstimulated whole salivary flow (UWSF), **(C)** stimulated whole salivary flow (SWSF), and **(E)** tear secretion (Schirmer’s test) were significantly reduced after treatment compared with baseline. **(D)** Intraoral photographs of patients with xerostomia. Data represent mean ± SEM. Paired t-test; *p < 0.05, **p < 0.01, ***p < 0.001.

The study cohort comprised 25 pathologically confirmed patients (21 male; median age 52 years) with HNSCC who were being treated with pembrolizumab or tislelizumab, a biologic targeting programmed cell death 1 (PD‐1) (**Figure 1A**). No patient had a pre‐existing autoimmune disease. The median interval between the onset of ICI and the onset of dry mouth was 3 months (**Table 1**).

**Table 1:** Baseline characteristics of patients.

Patient	Gender	Age at diagnosis	Previous autoimmune	Underlying cancer	Tumor classification	Previous history	Smoking history	Drinking history	Chewing betelnut history
1	Male	35	No	Right tongue	cT3N1M0	Hepatitis B, Nasopharyngeal carcinoma	Yes	Yes	No
2	Male	69	No	bilateral mouth floor	cT4aN2bM0	No	Yes	Yes	No
3	Female	58	No	Left inferior gingiva	cT3N2bM0	No	No	No	No
4	Male	52	No	Left buccal	cT3N2bM0	No	No	No	No
5	Male	36	No	Right tongue	cT4aN2bM0	No	Yes	Yes	Yes
6	Male	47	No	bilateral maxillary sinus	cT4aN2bM0	Diabetes Mellitus, Hyperuricemia	Yes	Yes	No
7	Male	57	No	Right tonsil, root of tongue	cT4bN3bM0	Hypertension, Diabetes Mellitus	No	Yes	No
8	Male	57	No	Left skull base, maxilla	cT4bN0M0	Diabetes Mellitus	Yes	No	No
9	Female	25	No	Right mouth floor, tongue	cT3N1M0	No	No	No	No
10	Male	44	No	Left palate	cT4aN0M0	No	No	No	No
11	Male	56	No	Right palate, Right oropharynx , bilateral tongue	cT4aN3bM0	No	Yes	Yes	Yes
12	Female	65	No	bilateral upper lip, superior gingiva	cT3N0M0	No	No	No	No
13	Male	65	No	Right maxilla, gingiva, oropharynx	cT4bN0M0	Hypertension	Yes	Yes	Yes
14	Male	49	No	bilateral tongue, mouth floor	cT4aN2aM0	No	Yes	Yes	No
15	Male	48	No	bilateral tongue	cT3N0M0	No	No	Yes	No
16	Male	43	No	Left mouth floor, buccal, inferior gingiva	cT4aN0M0	Hypertension	Yes	Yes	Yes
17	Male	58	No	Right mouth floor, tongue	cT2N2bM0	No	Yes	Yes	Yes
18	Male	67	No	Right superior gingiva	cT3N0M0	Hypertension, Diabetes Mellitus	No	No	No
19	Female	75	No	Left buccal	cTisN0M0	Breast cancer, Hypertension, Diabetes Mellitus	No	No	No
20	Male	50	No	Right tongue	cT3N0M0	No	No	No	Yes
21	Male	61	No	bilateral inferior gingiva	cT4aN2bM0	Hypertension	Yes	Yes	Yes
22	Male	59	No	Left root of tongue, oropharynx	cT4aN3bM0	Hypertension, Diabetes Mellitus	Yes	Yes	No
23	Male	73	No	Right inferior gingiva, buccal	cT4bN1M0	Hypertension, Diabetes Mellitus	No	Yes	No
24	Male	32	No	Right tongue	cT3N0M0	No	Yes	Yes	Yes
25	Male	52	No	Left oropharynx	cT4bN0M0	Hypertension	Yes	Yes	Yes

To quantitatively assess the impact of ICI therapy on exocrine gland function, we prospectively monitored salivary and lacrimal secretion in the 25 patients before and after treatment. After treatment, the total unstimulated salivary flow (UWSF) was significantly reduced compared with the pre-treatment levels (*P <* 0.05), with decreased secretion observed in 64% of patients (16/25) (**Figure 1B**). Notably, all treated patients exhibited UWSF rates below the diagnostic threshold for hyposalivation (1.5 mL/15 min), with a median of 0.45 mL/15 min (range: 0–2.49 mL/15 min), indicating severe resting-state salivary hypofunction. Similarly, Stimulated Whole Salivary Flow (SWSF) following exposure to 3% citric acid exhibited significant post-treatment reduction in 72% of patients (18/25) (*P <* 0.05) (**Figure 1C**). A common clinical sign among the enrolled patients was severe xerostomia, characterized by the absence of salivary pooling and an atrophic tongue surface (**Figure 1D**). Beyond salivary dysfunction, lacrimal gland function, assessed by the Schirmer’s test, was also significantly impaired. Post-treatment tear secretion demonstrated marked bilateral reduction compared with baseline measurements in 68% of patients (17/25) (*P <* 0.05), providing an objective physiological basis for xerophthalmia (**Figure 1E**).

From a subjective assessment perspective, we employed the Multidisciplinary Salivary Gland Society (MSGS) scale (30) as our primary evaluation tool. Despite the lack of significant changes in xerostomia scores on the MSGS scale (**Supplementary Figure S1**), subsequent clinical interview assessments revealed a substantial symptomatic burden reported by patients. Xerostomia was often exacerbated during physical activity or at night, and some individuals experienced nocturnal awakening due to tongue–palate adhesion, which required frequent water intake. Additional complaints included viscous saliva, dry throat with hoarseness, dysgeusia (altered taste perception), and reduced tolerance to spicy or acidic foods.

### 2.2 ICI therapy caused salivary gland fibrosis and destruction of functional acinar structures

**Figure 2:**
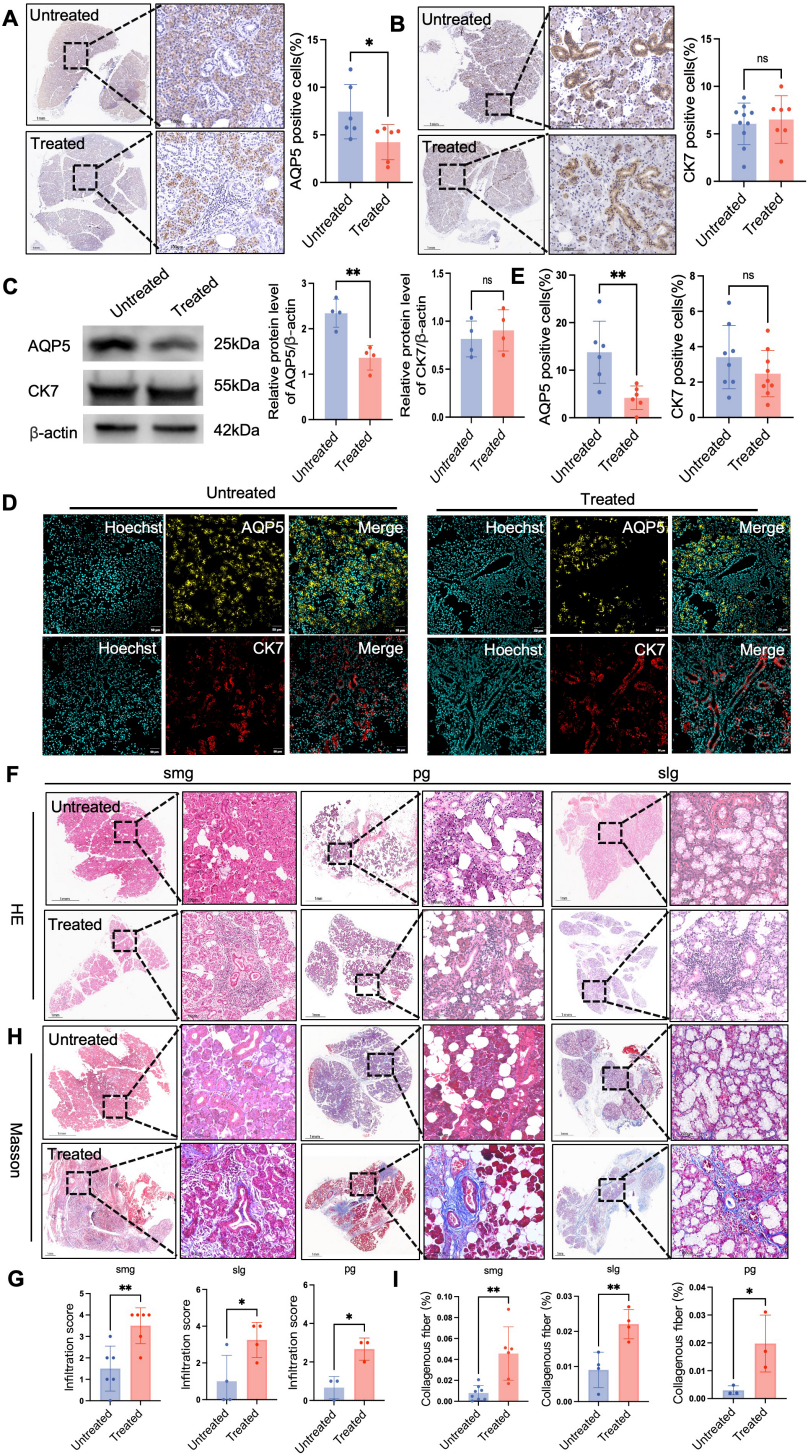
ICI therapy causes salivary gland fibrosis and destruction of functional acinar structures. **(A)** Immunohistochemistry (IHC) for aquaporin-5 (AQP5) in salivary gland (SG) sections from control and treated patients; nuclei counterstained with hematoxylin. Scale bars, 1 mm (1×) and 100 µm (20×). Right: quantification as percent AQP5-positive area per field. **(B)** IHC for cytokeratin 7 (CK7); nuclei counterstained with hematoxylin. Scale bars, 1 mm (1×) and 100 µm (20×). Right: quantification as percent CK7-positive area per field. **(C)** Western blots of AQP5 and CK7 in SG lysates; β-actin as loading control. Right: densitometry normalized to β-actin. **(D)** Immunofluorescence for AQP5 (red), CK7 (yellow), and nuclei (Hoechst, blue). Scale bars, 20 µm. **(E)** Quantification of mean fluorescence intensity (MFI) for AQP5 and CK7. **(F)** Hematoxylin and eosin (H&E) staining of submandibular (SMG), parotid (PG), and sublingual (SLG) glands. Scale bars, 1 mm (1×) and 100 µm (20×). **(G)** Lymphocytic infiltration quantified by focus score (number of foci ≥50 lymphocytes per 4 mm^2). Data are mean ± SEM. Mann–Whitney U test. **(H)** Masson’s trichrome staining of SMG, PG, and SLG; collagen blue, cytoplasm/muscle red. Scale bars, 1 mm (1×) and 100 µm (20×). **(I)** Fibrotic area quantified as percent blue-stained area per field. Data are mean ± SEM. Student’s t test. *p < 0.05, **p < 0.01.

To elucidate the pathological basis of glandular hypofunction after ICI therapy, we analyzed salivary gland specimens (submandibular gland, SMG; sublingual gland, SLG; and parotid gland, PG) from 50 patients treated with and without ICI. The baseline information is shown in **Table 2**. The acinar marker aquaporin-5 (AQP5) (31) and the ductal marker cytokeratin 7 (CK7) were analyzed, reflecting secretory function and ductal architecture, respectively. The ICIs-treated group and the untreated group showed different patterns: In the untreated group, AQP5 and CK7 precisely delineated the acinar and ductal structures (**Figures 2A, B**). In contrast, glands from the ICIs group exhibited widespread atrophy and loss of AQP5-positive acini (**Figures 2A, C, D**). Quantitative analysis confirmed a significant downregulation of the expression of the AQP5 protein in the ICI group compared with that of controls, whereas the CK7 protein remained unchanged (**Figure 2E**). These findings suggest that ICI therapy causes selective damage to acinar cells, thereby compromising glandular secretory function, while the ductal structures remained relatively intact.

**Table 2:** Baseline characteristics of patients whom clinical specimens were obtained.

Characteristic	Untreated immunotherapy group (n=20)	Treated immunotherapy group (n=30)	Total(n=50)
Year	50.7 ± 15.0	51 ± 12.75	
Sex			
Male	14	25	39
Female	7	4	11
Position of tumor			
Tongue	15	13	28
Buccal	2	6	8
Gingival	2	5	7
other	1	6	7
Smoking history			
+	10	24	34
-	11	5	16
History of chewing betelnut			
+	7	11	18
-	14	18	32
History of alcoholism			
+	9	22	31
-	12	7	19
Radiotherapy			
+	0	0	0
-	21	29	50
Chemotherapy history			
+	1	3	4
-	20	26	46
TNM stage			
I	2	0	2
II	3	0	3
III	9	6	15
IVA	7	13	20
IVB	0	8	8
IVC	0	2	2
Comorbidities			
Hypertension	5	3	8
Diabetes Mellitus	3	1	4

The destruction of the acinar and duct appeared to be the result of lymphocyte infiltration. A striking example was observed in a patient who had received five cycles of ICI therapy, and whose submandibular gland showed near-total acinar destruction, with remnant ducts engulfed by diffuse lymphocytic infiltrates (**Figure 2D**). Hematoxylin and Eosin (H&E) staining revealed prominent focal lymphocytic infiltrates in glands of the ICI group, predominantly in periductal areas, which were absent in controls (**Figure 2F**). Semi-quantitative scoring confirmed this finding (*P <* 0.05) (**Figure 2G**).

To assess tissue remodeling, Masson’s trichrome staining was performed. This revealed extensive deposition of dense collagen fibers, indicative of significant fibrosis, in the interlobular and periductal stroma of the ICI group, a feature not observed in controls (**Figures 2H, I**).

Collectively, these findings demonstrate that ICI therapy induces periductal-centric lymphocytic sialadenitis accompanied by severe fibrosis. This pathological process culminates in the destruction of functional acinar-ductal units, which is responsible for the progressive loss of secretory function.

### 2.3 Immunotherapy-induced sialadenitis was characterized by a unique CD4^+^ T cell-dominant lymphocytic infiltrate

**Figure 3:**
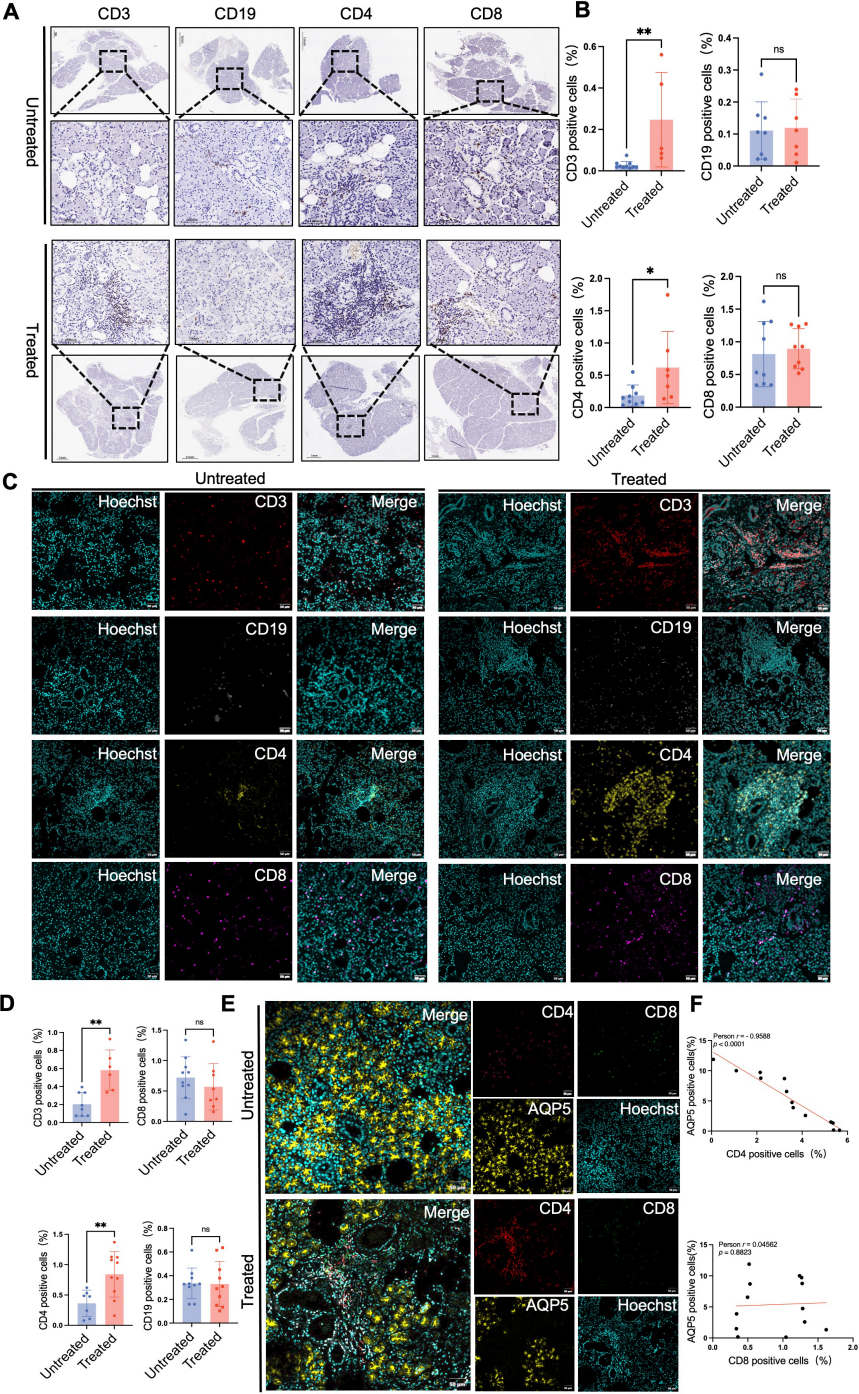
Immunotherapy-induced sialadenitis is characterized by a unique CD4^+^ T cell-dominant lymphocytic infiltrate. **(A)** Representative immunohistochemical (IHC) staining for CD3 (pan T-cell marker), CD19 (B-cell marker), CD4 (T-helper cell marker), and CD8 (cytotoxic T-cell marker) in salivary gland tissues. Positive staining is indicated by brown diaminobenzidine (DAB), and sections were counterstained with hematoxylin (blue).Scale bars, 1 mm (1×) and 100 μm (20×). **(B)** Quantitative analysis of the number of CD3^+^, CD19^+^, CD4^+^, and CD8^+^ positive cells per mm² of tissue. **(C)** Immunofluorescence showing organized lymphocytic foci in the treatment group, with clusters of CD19^+^ (white) surrounded by CD3^+^ (red), including both CD4^+^(yellow) and CD8^+^ subsets (pink). Nuclei are stained blue with Hoechst. Scale bars, 50 μm. **(D)** Quantitative spatial analysis of immune cell composition and density (cells per mm^2). Data are mean ± SEM. Student’s t test or Mann–Whitney U test; *p < 0.05, **p < 0.01. **(E)** Representative multiplex immunofluorescence images showing CD4^+^ (red), CD8^+^ (green), and AQP5^+^(yellow) staining in submandibular glands. Nuclei are stained blue with Hoechst. Scale bars, 50 µm. **(F)** Correlation plots of T cell subset density versus AQP5^+^ area percentage. A significant negative correlation was found for CD4^+^T cells, while the correlation was not significant for CD8^+^ T cells. Statistics were calculated using Spearman’s correlation. Spearman’s correlation (ρ) and p-values are shown.

The pathological profile of sialadenitis is etiology-dependent and requires a careful differential diagnosis. Therefore, to identify the underlying cause of this ICI-induced-condition, further elucidation of its pathological characteristics is required. To characterize the immune composition of ICI-induced sialadenitis, we performed immunohistochemistry and immunofluorescence staining of tissue sections in 3 major glands. The results revealed that lymphocytic foci were densely populated by CD3^+^ T cells and CD19^+^ B cells were scattered only sparsely and individually, failing to form aggregates (**Figures 3A, B**).

**Figure 4:**
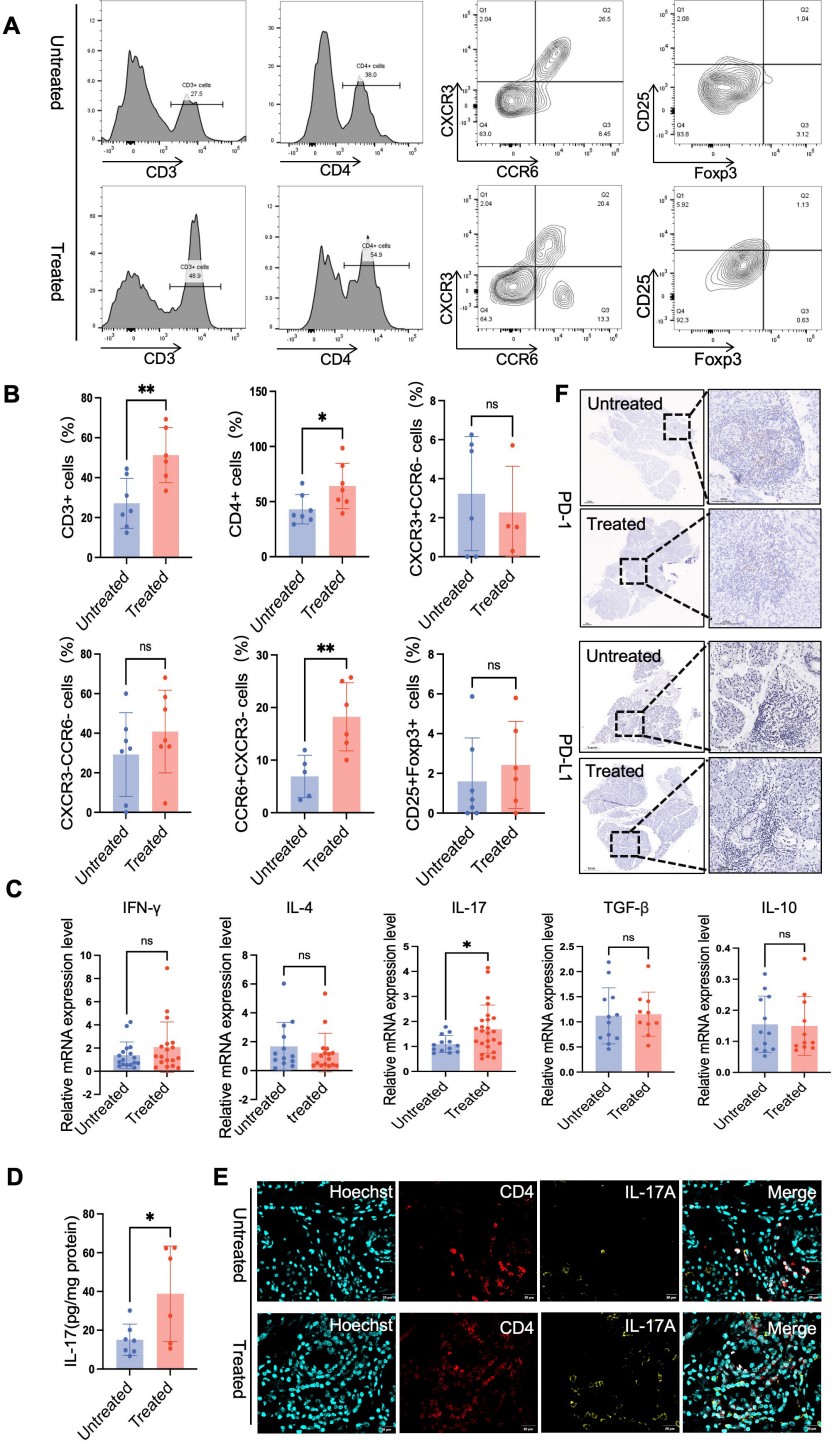
The Th17/IL-17 axis is the core pathogenic pathway driving immunotherapy-induced sialadenitis. **(A)** Representative flow cytometry plots illustrating the gating strategy for immunophenotyping of infiltrating leukocytes from dissociated salivary gland tissues. Live, single cells were first isolated, followed by the identification of CD45^+^ cells (leukocytes). Within this population, T cells were defined as CD3^+^ cells. T cells were further resolved into CD4^+^ and CD8^+^ subsets. The CD4^+^ T-helper cell compartment was subsequently analyzed for the expression of chemokine receptors CXCR3 and CCR6 to identify Th1 (CXCR3^+^CCR6^-^), Th2 (CXCR3^-^CCR6^-^), and Th17 (CCR6^+^CXCR3^-^) subsets. Using the identical initial gating hierarchy, Regulatory T cells (Tregs) were identified as CD25^+^FOXP3^+^. **(B)** Quantification of the frequencies of major immune cell populations in control and treatment groups. Bar graphs show the percentages of T cells (within the CD45^+^ gate), CD4^+^ and CD8^+^ T cells (within the CD3+ T-cell gate), and Th1, Th2, Th17 cells and Treg cells (within the CD4^+^ T-cell gate). **(C)** Quantitative real-time PCR (qRT-PCR) analysis of signature cytokine gene expression in CD4+ T cells purified from the salivary glands of control and treated patients. The expression of IFNG (Th1 signature), IL4 (Th2 signature), IL10, and TGFB1 (Treg/immunosuppressive signature) was assessed. Gene expression levels were normalized to the endogenous control GAPDH. Data are presented as relative fold change compared to the control group, calculated using the 2^-ΔΔCt method. **(D)** IL-17A protein levels in salivary gland tissue lysates were measured by ELISA. The concentration of IL-17A was normalized to the total protein content of each sample. **(E)** Immunofluorescence for CD4 (red) and IL-17A (green) in salivary glands. Co-expressing Th17 cells (yellow) were abundant in the treatment group but rare in controls. Nuclei are stained with Hoechst (blue). Scale bar, 50 μm. **(F)** IHC staining of PD-1 and PD-L1 in salivary glands. Scale bars,1mm(1×) and 100μm(20×).Results are shown as mean ± SEM. Statistical significance was determined using Student’s t-test or Mann-Whitney U test. *p < 0.05, **p < 0.01.

Further analysis of T cell subsets revealed that the distribution of CD4^+^ T cells within the foci closely mirrored that of CD3^+^ T cells, identifying them as the core component of the infiltrated lymphocyte. Compared with the untreated group, the glands of the ICI group showed a significant increase in the density of CD3^+^ and CD4^+^ T cells (*P <* 0.05). However, the densities of CD8^+^ T cells and CD19^+^ B cells did not differ significantly between groups. Moreover, although CD8^+^ cytotoxic T cells were present, they were diffusely scattered and did not form dominant clusters within the foci (**Figure 3C**). Quantitative analysis corroborated these observations (**Figure 3D**). To identify the immune cell population driving glandular damage, we conducted multiplex immunofluorescence which revealed a striking spatial association: dense CD4^+^ T cell infiltrates consistently corresponded with a profound loss of AQP5^+^ acini. This association was not apparent for CD8^+^ T cells (**Figure 3E**). Quantitative analysis confirmed this observation, demonstrating a significant negative correlation between CD4^+^ T cell density and AQP5-positive area (Spearman’s ρ = -0.9588, P < 0.0001) (**Figure 3F**). These findings pinpoint these CD4^+^ T cells as the primary mediators of acinar destruction in this disease. To support our histological findings and quantitatively define the infiltrating leukocyte populations, we performed flow cytometry on single-cell suspensions isolated from salivary gland specimens (**Figure 4A**). Due to the limited availability of patient samples, the samples were restricted to the submandibular gland. In particular, the submandibular gland contributes about 70% of total saliva secretion, highlighting its predominant role in salivary function and the rationale for focusing our analysis on this gland. Consistent with our IHC data, flow cytometric analysis revealed a significant increase in the percentage of CD3^+^ and CD4^+^ T cells in the ICI group compared with that of controls (*P <* 0.05). (**Figure 4B**).

Therefore, immunotherapy-induced sialadenitis presents a unique immune profile: a CD4^+^ T cell-driven, B cell-quiescent, non-myeloid adaptive immune response.

### 2.4 The Th17/IL-17 axis was the core pathogenic pathway driving immunotherapy-induced sialadenitis

Given the predominance of CD4^+^ T cells, we further examined their main functional subsets, including Th1, Th2, Th17, and regulatory T cells (Tregs) (**Supplementary Figure S2**). The markers (Th1: CXCR3^+^CCR6^-^; Th17: CCR6^+^ CXCR3^-^; Th2: CXCR3^-^CCR6^-^; Treg: CD25^+^Foxp3^+^) provided critical insight. Among all the subsets of CD4^+^ T cells analyzed, only the population of Th17 cells was significantly and specifically expanded in the ICI group (*P <* 0.05) (**Figure 4B**). In contrast, the proportions of Th1, Th2, and Treg cells did not differ between the groups (**Figure 4B**).

To determine whether this expansion of Th17 cells was functionally relevant, we assessed the expression of their hallmark effector cytokine, IL-17A, at the transcript, protein and tissue levels. First, quantitative real-time PCR (qPCR) analysis revealed a significant upregulation of IL17A mRNA in the ICI group (*P <* 0.05) (**Figure 4C**). In contrast, the signature cytokine mRNA levels for Th1 (IFN-γ), Th2 (IL-4), and Tregs (IL-10, TGF-β) were comparable between the groups (**Figure 4C**). This finding was corroborated at the protein level by ELISA, which demonstrated significantly higher concentrations of secreted IL-17A in tissue homogenates from the ICI group (*P <* 0.05) (**Figure 4D**). Finally, to link IL-17A production with infiltrating lymphocytes *in situ*, we performed immunofluorescence staining. The imaging revealed a clear co-localization of the IL-17A protein with infiltrating CD4^+^ T cells, confirming these cells as the primary source of cytokines within the tissue (**Figure 4E**). Nevertheless, given the therapeutic target of ICIs, we examined the expression of programmed death-1/programmed death-ligand 1 (PD-1/PD-L1). Immunohistochemical analysis revealed the absence of PD-L1 in normal ductal and acinar epithelial cells. Within inflammatory infiltrates, although some lymphocytes showed positive PD-1 staining, the intensity was generally mild to moderate (**Figure 4F**). Furthermore, the staining for myeloid cell markers (e.g., macrophages and neutrophils) was negative, indicating that the infiltrate was not driven by innate immune cells (**Supplementary Figure S3**).

### 2.5 A Preclinical model recapitulates the clinical phenotype and reveals a therapeutic role for IL-17A blockade

**Figure 5:**
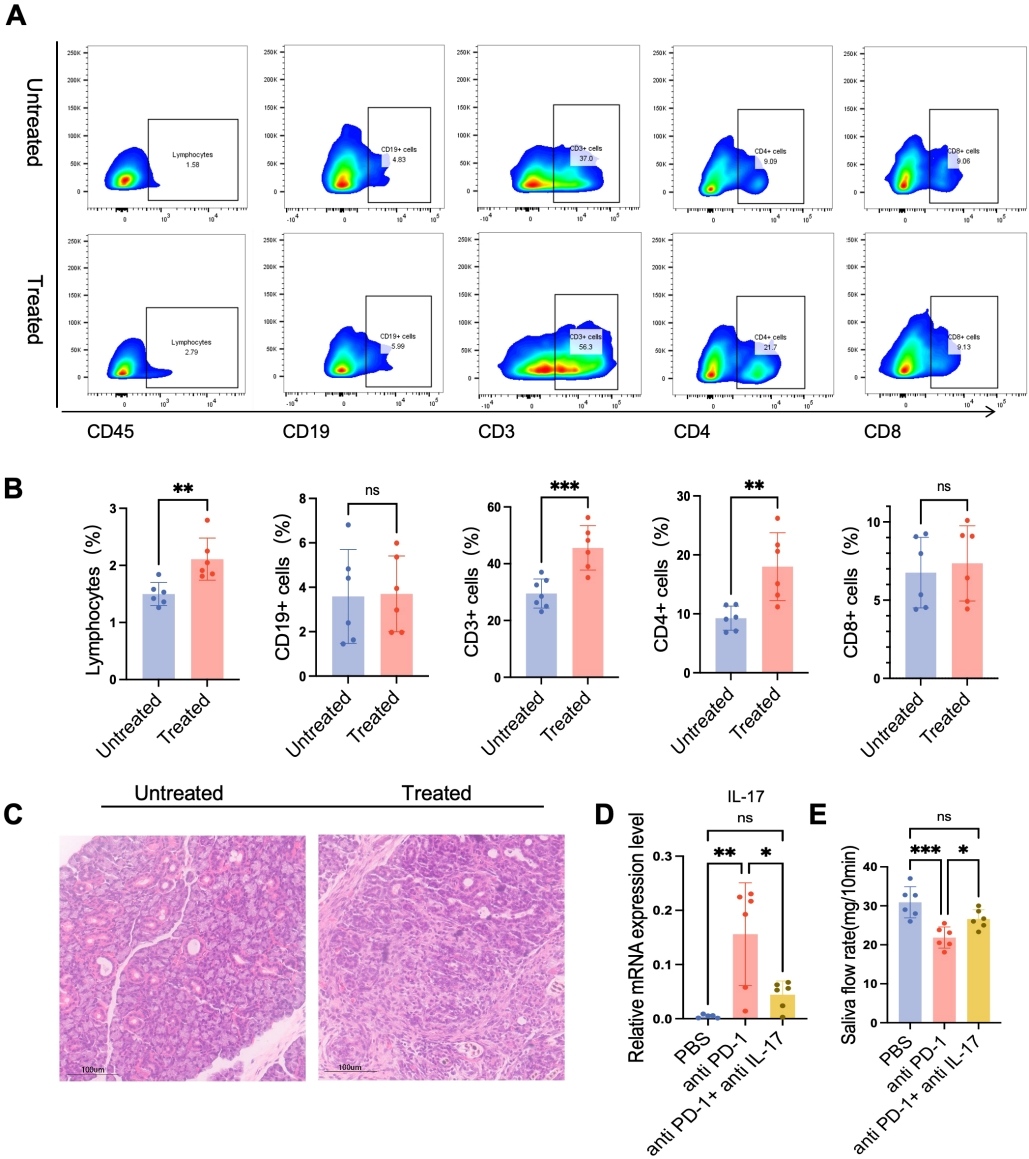
IA preclinical model recapitulates the clinical phenotype and reveals a therapeutic role for IL-17A blockade. **(A, B)** Representative flow cytometry plots illustrating the gating strategy for immunophenotyping of infiltrating leukocytes from dissociated salivary gland tissues. Live, single cells were first isolated, followed by the identification of CD45^+^ cells (leukocytes). Within this CD45^+^ population, T cells were defined as CD3^+^ and B cells as CD19^+^. T cells were further resolved into CD4^+^ and CD8^+^ subsets. Bar graphs show the percentages of T cells and B cells (within the CD45^+^ gate), as well as CD4^+^ and CD8^+^ T cells (within the CD3^+^ T-cell gate). **(C)** Representative Hematoxylin and Eosin (H&E) stained sections of submandibular glands (SMGs) from a control mouse and a mouse treated with an anti-PD-1 antibody. Scale bars, 100 µm. **(D)** Quantitative RT-PCR analysis of IL-17 mRNA expression in SMG tissue from three experimental groups: Control, anti-PD-1 + Isotype control, and anti-PD-1 + anti-IL-17A antibody. Data were normalized to the expression of the housekeeping gene Gapdh and are presented as fold change relative to the Control group. **(E)** Measurement of pilocarpine-stimulated salivary flow rates from the three indicated treatment groups. Saliva was collected over a defined time interval, and its total weight was measured. Data are presented as flow rate in milligrams per minute (mg/10min). Statistical significance was determined using Student’s t-test or Mann-Whitney U test. *p < 0.05, **p < 0.01, ***p < 0.001.

To establish a preclinical model that recapitulates our clinical findings, we administered an anti-PD-1 antibody to mice bearing tongue squamous cell carcinoma. Consistent with our observations in patient biopsies, flow cytometric analysis of SMGs from these mice revealed a significant increase in infiltrating CD45^+^ leukocytes. Within this CD45^+^ population, we observed a significant expansion of the CD3^+^ T cell compartment, with no change in B cell frequency. Furthermore, the T cell population displayed a pronounced skewing towards a CD4^+^ dominant phenotype (**Figures 5A, B**). Histopathological examination further confirmed this parallel, showing massive lymphocytic infiltration and severe destruction of acinar architecture, mirroring the pathology seen in our patient cohort (**Figure 5C**).

Given the CD4^+^ T cell dominance, we hypothesized that IL-17A plays a key role in this process. Indeed, anti-PD-1 treatment led to a robust upregulation of Il17a gene expression in the SMGs (**Figure 5D**) and a concomitant, significant reduction in salivary flow (**Figure 5E**). To test the functional relevance of this pathway, we administered a neutralizing anti-IL-17A antibody. This intervention not only attenuated the upregulation of IL-17A in the gland tissue but also led to a significant recovery of saliva production compared to the anti-PD-1 group (**Figures 5D, E**). Taken together, these data establish a causal link between PD-1 blockade, IL-17-mediated immunopathology, and salivary gland dysfunction, highlighting the IL-17 axis as a promising therapeutic target to ameliorate this immune-related adverse event.

In conclusion, these findings demonstrate a selective expansion of Th17 cells accompanied by enhanced IL-17A production, implicating this pathway as a key mediator of ICI-induced salivary gland inflammation.

## 3 Discussion

This study provides a comprehensive characterization of ICI-induced sialadenitis, bridging clinical observations with a preclinical model that not only recapitulates the human immunopathology but also validates a key molecular driver as a druggable therapeutic target. Our results indicate that activation of the Th17/IL-17 signaling axis may serve as a pathogenic mechanism that underlies this condition. Collectively, these findings support the view that ICI-induced sialadenitis should be viewed as a distinct pathological entity, rather than merely a subtype of SjS.

We documented significant reductions in salivary and lacrimal secretion after ICI treatment, measured by UWSF/SWSF and Schirmer’s tests. Nearly all patients developed hyposalivation, consistent with previous reports showing 96% incidence of xerostomia following PD-1/PD-L1 inhibitor treatment (32). Surprisingly, however, the MSGS scale (30) assessment showed no significant deficits. This may be attributed to the patients’ prior awareness of side effects and their tumor-bearing status, which might have led to reduced attention to xerostomia symptoms. Furthermore, histopathological analysis revealed distinctive glandular damage patterns, characterized by acinar and ductal destruction, immune cell infiltration, and fibrosis in ICI-treated specimens. To uncover the mechanism to the damage of gland tissue, we further analyze the compartment of immune infiltration. ICI-induced sialadenitis exhibited a distinctive CD4^+^ T cell-dominant, B cell-quiescent immune profile, consistent with the mechanism of ICIs in reversing T-cell exhaustion (33). Consistent with our results, Warner et al. and Pringle, et al. found that salivary gland biopsies showed mainly T-cell infiltration, with CD4^+^ cells slightly exceeding CD8^+^, and only sparse CD20^+^ B cells (16) (34). This immunophenotype differs from SjS, which is characterized by B-cell hyperactivity (24), germinal center formation, and autoantibody production (35), as well as with IgG4-related disease, which is defined by dense IgG4^+^ plasma cell infiltration and storiform fibrosis (36, 37). However, some studies have reported contrasting findings. A recent study by Borys et al. in a murine model of ICI therapy reported an abundance of highly activated CD8^+^ T cells rather than CD4^+^ T cells in the submandibular gland (38). The differences may reflect variations in ethnic background (39), ICI drug type (40), induction models (41–44), target organs (45–48) or individual susceptibility (49–51). Collectively, our findings provide both clinical evidence of ICI-induced exocrine dysfunction and establish distinct pathological criteria for differential diagnosis of ICI-induced sialadenitis. We postulated that in the context of ICI-mediated disruption of peripheral tolerance, salivary gland damage follows a pathogenic cascade driven by the Th17/IL-17 axis. By reversing T-cell exhaustion and restoring cytotoxic function, proliferation, and cytokine production, ICIs effectively remodel the immunosuppressive tumor microenvironment (TME) and enhance endogenous anti-tumor immunity (33, 52). However, this systemic immune activation can inadvertently disrupt immune tolerance in healthy tissues (48). Although clinical (44, 48, 53) and preclinical studies (41–43) have established that T-cell activation is a hallmark of ICI-related adverse events (47), the specific T-cell subsets involved in mediating tissue-specific immune injury remain largely undefined (54, 55) (56). A key finding of our study is that this activation appears not to be random but instead skews naïve T cells toward the Th17 lineage. Prior research has shown that several irAEs, including colitis and dermatitis, are driven by T-cell activation and augmented Th1/Th17 responses (57–59) (56). However, we found that weak PD-1 immunoreactivity and lack of PD-L1 expression in the salivary glands indicate that these tissues may not be direct targets of ICI therapy. Together with our results, we hypothesize a possible sequential pathogenic process: ICI treatment first triggers immune activation at primary tumor sites, followed by migration of activated lymphocytes to the salivary glands through epitope spreading mechanisms. This “off-target” immune response may explain how ICI therapy leads to sialadenitis as a secondary manifestation of systemic immune activation. Within the salivary gland, expanded Th17 cells release their hallmark cytokine IL-17A (60) (61), thereby initiating a cascade of downstream tissue-destructive events. First, IL-17A promotes periductal lymphocytic aggregation (62), resembling early tertiary lymphoid structures (63–65), by upregulating adhesion molecules (66) and chemokines (CXCL13, CCL19, CCL21) (67), thereby recruiting and retaining immune cells within an inflammatory milieu enriched in TNF-α (68), IL-1β, and IL-6 (69). Second, it drives progressive fibrosis by activating fibroblasts (70) and inducing epithelial–mesenchymal transition (71–73) in ductal cells, leading to the generation of myofibroblasts that produce excess extracellular matrix and increase TIMPs expression (74), resulting in collagen accumulation and dense periductal fibrosis (75) that replaces functional parenchyma and obstructs residual ducts and acini (76–78). Finally, IL-17A exerts direct and indirect damage on epithelial cells (77, 78), culminating in a selective destruction of acini while ductal structures are preserved (79), which explains the severe reduction of unstimulated salivary flow predominantly dependent on acinar function. We propose that this selectivity is driven by a combination of factors: the preferential targeting of acinar cells by the immune system, their limited regenerative capacity relative to ductal progenitors (80), and their intrinsic vulnerability as high-metabolism secretory cells. (81, 82)To our knowledge, this is the first study to specifically target major salivary gland biopsy, whereas most previous research has mainly focused on a minor salivary gland biopsy (18, 25). Considering that the major salivary glands are responsible for approximately 99% of the total daily salivary output (83), their functional and pathological alterations are likely to have a much greater impact on oral homeostasis (14, 15, 84). Therefore, elucidating the changes occurring in the major salivary glands following ICI therapy is of particular importance for understanding the pathogenesis of ICI-associated sicca manifestations and for guiding potential therapeutic strategies. In addition to qualitative immunohistochemistry (IHC) analysis, we quantitatively assessed immune cell proportions, which demonstrated the predominance of Th17 cells in ICI-treated salivary glands. These findings offer a rationale for exploring targeted cytokine therapies as a potential approach to mitigate irAEs. For instance, anti-IL-17A antibodies, such as eculizumab, have been successfully employed to treat other Th17-driven irAEs, including arthritis (48) and dermatitis (85). Furthermore, in murine models of ICI therapy, antibodies targeting IL-25 (IL-17E) or the IL-17 receptor (IL-17RA) have been shown to inhibit the development of pneumonitis and hepatitis while exhibiting additional anti-tumor activity (42, 86, 87). To validate our clinical findings, we successfully established a preclinical murine model of HNSCC undergoing anti-PD-1 therapy. These mice developed sialadenitis characterized by histopathological changes and lymphocytic infiltration in the submandibular glands that mirrored those observed in our patient cohort. Critically, therapeutic blockade of IL-17A in this model led to a significant restoration of salivary flow, providing direct evidence for the pathogenic role of the IL-17 axis and its potential as a therapeutic target. Our research uncovers the potential for such agents as effective treatments, and potentially preventing severe immunotherapy-induced sialadenitis, thereby significantly improving patient quality of life without compromising anti-tumor efficacy.

Some limitations of this study should be acknowledged. First, the sample size was relatively small and our findings require validation in larger multicenter cohorts. The lack of serological testing did not allow us to fully understand systemic changes in ICIs treated patients, which might have provided a better understanding of similarities and differences between SjS and ICI-induced sialadenitis. Second, although we have established the central role of the Th17/IL-17 axis, upstream signals, such as why T cells preferentially polarize toward the Th17 lineage within the salivary gland, remain to be elucidated. Finally, our primary focus was alleviating sialadenitis, and we did not assess the potential interference of IL-17 blockade with ongoing anti-tumor immunotherapy. Given the pleiotropic nature of IL-17 in cancer, decoupling irAE management from anti-tumor efficacy presents a significant translational hurdle. Future research is strictly required to define a therapeutic window that alleviates sialadenitis without compromising tumor control.

## 4 Conclusion

By integrating direct analysis of human specimens with a functionally validated preclinical model, this study establishes the Th17/IL-17 axis as a causal pathogenic driver of immunotherapy-induced sialadenitis. These findings provide a strong rationale for refining diagnostic criteria and, more importantly, offer the first preclinical proof-of-concept for a targeted therapeutic strategy to manage this condition.

## 5 Material and methods

### 5.1 Study participants

We prospectively enrolled patients 18 years or older with a confirmed diagnosis of HNSCC who were treated with pembrolizumab and/or nivolumab at our institution between 2023 and 2025. Exclusion criteria included (1): concomitant radiotherapy (2); a history of specific chronic sialadenitis, such as SjS or IgG4-related disease; and (3) concurrent use of medications known to significantly affect salivary or lacrimal function. The institutional ethics committee approved the study protocol and all amendments of the Hospital of Stomatology of Sun Yat-sen University, China (Grant Number: ERC- [2015]-29) in accordance with the Declaration of Helsinki. Written informed consent was obtained from the patients for the publication of any potentially identifiable images or data included in this article.

### 5.2 Salivary gland tissue collection and processing

Salivary gland tissue samples were obtained intraoperatively from patients who underwent cervical lymph node dissection as part of their surgical treatment. Immediately after excision, each fresh specimen was divided. One portion was placed in ice-cold RPMI 1640 medium and transported on ice for subsequent experiments. Another portion was fixed in 4% paraformaldehyde for 24–48 h at 4°C, followed by standard processing for dehydration and paraffin embedding. The resulting paraffin-embedded blocks were sectioned into 4 μm-thick slices, which were then mounted on glass slides for subsequent analysis. A third portion was snap-frozen in liquid nitrogen and stored at -80°C for future molecular analyses.

### 5.3 Clinical assessments of salivary and lacrimal function

All functional assessments were performed at the beginning of the study (before ICI treatment) and at designated follow-up time points. To minimize diurnal variations, saliva samples were collected between 9:00 a.m. and 11:00 a.m. Patients were instructed to fast for at least one hour prior to collection. The flow rate of unstimulated whole saliva (UWS) was measured using the passive drool method over a 15-min period in a pre-weighed sterile tube. Subsequently, the flow rate of the stimulated whole saliva (SWS) was assessed for 15 min, and salivary secretion was stimulated by applying 3% citric acid to the tongue tip every 3 min. Both UWS and SWS flow rates were calculated and expressed in mL/min. Lacrimal secretion was quantified using the standard Schirmer’s test. A sterile filter paper strip was placed in the lower conjunctival fornix of each eye for 5 min. The length of the moistened portion of the strip was measured in millimeters (mm).

### 5.4 Histological staining

Before staining, paraffin-embedded sections (4 μm) were deparaffinized and rehydrated. Briefly, slides were baked at 65°C for 15 min, immersed in xylene, and then passed through a gradient of ethanol. (100%, 95%, 80%, 70%) to distilled water. H&E staining was performed using a commercial kit (Solarbio, Cat# G1120), following the manufacturer’s instructions. Masson’s trichrome staining was conducted similarly (Solarbio, Cat# G1340). Following staining, all sections were dehydrated through a graded ethanol series, cleared in xylene, and cover slipped with a neutral mounting medium. H&E-stained sections were used for histopathological assessment. The severity of lymphocytic infiltration in salivary glands was graded according to the Chisholm and Mason scoring system as follows: Grade 0: no inflammatory infiltrate; Grade 1: mild, diffuse lymphocytic/plasmocytic infiltrate; Grade 2: moderate infiltrate or less than one focus; Grade 3: one focus; Grade 4: more than one focus. A “focus” was defined as an aggregate of at least 50 mononuclear cells per 4 mm² of tissue. For morphometric analysis, whole-slide images (WSIs) were acquired using a digital slide scanner. Quantitative analysis was then performed on non-overlapping, randomly selected fields from each WSI using ImageJ software (NIH, USA).

### 5.5 Immunohistochemistry and immunofluorescence staining

Before staining, paraffin-embedded sections were deparaffinized in xylene and rehydrated through a graded ethanol series. Heat-induced epitope retrieval (HIER) was performed by incubating sections in sodium citrate buffer (10 mM, pH 6.0) in a pressure cooker for 5 min, followed by natural cooling to room temperature. Following antigen retrieval, endogenous peroxidase activity was quenched by incubating sections in 3% H_2_O_2_ for 15 min. After blocking non-specific binding, sections were incubated overnight at 4°C with the following primary antibodies: anti-AQP5(abcam ab315855), anti-CK7(abcam ab181598), anti-CD19(HUABIO ET1702-93), anti-CD3(Proteintech 17617), anti-CD4(ZSGB-BIO ZM0418), anti-CD8(ZSGB-BIO ZA0508), anti-CD68(CST 76437), anti-CD16(CST 24326),anti-PD-1(CST 86163),anti-PD-L1(CST 13684), and anti-IL-17A(abcam ab79056). The next day, sections were incubated with a horseradish peroxidase (HRP)-conjugated secondary antibody. The signal was visualized using a DAB (3,3’-diaminobenzidine) substrate kit. Finally, sections were counterstained with hematoxylin, dehydrated through a graded ethanol series, cleared in xylene, and cover slipped with a neutral mounting medium. For immunofluorescence staining, sections were blocked with 5% bovine serum albumin (BSA) for 60 min at room temperature. Sections were then incubated overnight at 4°C with the desired primary antibodies. After washing with PBST (PBS containing 0.1% Tween-20), sections were incubated with an Alexa Fluor 594-conjugated secondary antibody (1:1000) for 1 h in a dark, humidified chamber. Nuclei were counterstained with Hoechst. Slides were then mounted using an anti-fade mounting medium. Multiplex immunofluorescence staining was performed on 4-um FFPE sections using a commercial kit (Boster, Wuhan, China, Cat# PTSA-45) according to the manufacturer’s instructions. IHC-stained slides were digitized using a whole-slide scanner (Leica, Germany). Immunofluorescence images were captured using an Olympus motorized fluorescence microscope. Quantitative analysis was performed using ImageJ software (NIH, USA). For each marker, the extent of positive staining was quantified by calculating the percentage of the positively stained area relative to the total tissue area within each analyzed field.

### 5.6 Western blotting

Total protein was extracted from SG tissue using RIPA lysis buffer supplemented with a phosphatase and protease inhibitor cocktail. The lysates were cleared by centrifugation at 12,000 ×*g* for 15 min at 4°C, and the resulting supernatants were collected. The protein concentration was determined using a BCA Protein Assay Kit (Thermo Fisher Scientific).

For electrophoresis, equal amounts of protein (20 µg per lane) were resolved by SDS-PAGE and subsequently transferred to polyvinylidene difluoride membranes (PVDF) (Millipore, Billerica, MA, USA). Following transfer, the membranes were blocked with 5% non-fat dry milk in Tris-buffered saline containing 0.1% Tween-20 (TBST) for 1 h at room temperature. Membranes were then cut according to the expected molecular weights of the target proteins. The cropped membranes were incubated overnight at 4°C with specific primary antibodies: anti-AQP5, anti-CK7, anti-β-actin(Proteintech 66009). After washing with TBST, the membranes were incubated with appropriate HRP-conjugated secondary antibodies for 1 h at room temperature. Protein bands were visualized using an enhanced chemiluminescence (ECL) substrate (SuperSignal West Pico, Thermo Fisher Scientific) and imaged using a LI-COR Odyssey Clx Imaging System.

### 5.7 RNA extraction and quantitative real-time pcr

Total RNA was extracted from SG tissue samples using the FastPure^®^ Cell/Tissue Total RNA Isolation Kit V2 (Vazyme, Nanjing, China) according to the manufacturer’s protocol. The concentration and purity of the extracted RNA were determined using a spectrophotometer. First-strand cDNA was synthesized from total RNA using the HiScript^®^ III Reverse Transcriptase kit (Vazyme). RT-qPCR was performed using ChamQ Universal SYBR qPCR Master Mix¹ (Vazyme) on a QuantStudio™ 7 Flex Real-Time PCR System (Thermo Fisher Scientific, USA). The thermal cycling conditions were as follows: initial denaturation at 95°C for 5 min, followed by 40 cycles of 95°C for 15 s and 60°C for 60 s. A melt curve analysis was performed at the end of each run to confirm the specificity of the amplified products. The primers used for this study were: for human samples IL-17 5′-CTCTGTGATCTGGGAGGCAAA-3′ 5′- CTCTTGCTGGATGGGGACA-3′, IL-4 5′-CCAACTGCTTCCCCCTCTG-3′ 5′-TCTGTTACGGTCAACTCGGTG-3′, IL-10 5′-TCTCCGAGATGCCTTCAGCAGA-3′ 5′- TCAGACAAGGCTTGGCAACCCA-3′, IFN-Y 5’-TCGGTAACTGACTTGAATGTCCA-3’ 5’- TCGCTTCCCTGTTTTAGCTGC-3’, TGF-b 5’-GGCCAGATCCTGTCCAAGC-3’ 5’- GTGGGTTTCCACCATTAGCAC-3’, GAPDH 5′- GGAGCGAGATCCCTCCAAAAT-3′, and 5′- GGCTGTTGTCATACTTCTCATGG-3′. For mouse samples IL-17 5′- AAGCTGGACCACCACATGAA-3′ 5′- CCCTGAAAGTGAAGGGGCAG-3′, GAPDH 5′- AACTTTGGCATTGTGGAAGGG-3′, and 5′- GACACATTGGGGGTAGGAACA-3′.The relative expression levels of target genes were normalized to the endogenous control, GAPDH. The fold change in gene expression was calculated using the comparative Ct (2^-ΔΔCt^) method.

### 5.8 Enzyme-linked immunosorbent assay

The method for preparing proteins from salivary gland samples was the same as that described above. IL-17A concentration was quantified using a commercially available Human IL-17A ELISA kit (feiyue, FY-EH4665) following the manufacturer’s protocol. Briefly, absorbance was measured at 450 nm using a microplate reader (Biotek, USA). The concentration of IL-17A in each sample was calculated from a standard curve generated with recombinant IL-17A standards. The final results were normalized to the total protein content and are expressed as picograms of IL-17A per milligram of total protein (pg/mg).

### 5.9 ICIs tumor models

SCCVII cells were maintained in DMEM/F12 medium with 10% fetal bovine serum and 0.4% penicillin-streptomycin solution, which we obtained from the Peking University School and Hospital of Stomatology as a gift. To obtain SCCVII, a murine squamous cell carcinoma derived from immunocompetent mice C3H/HeN (C3H), we purchased female C3H mice (4–6 weeks of age) from Beijing Vital River Laboratory Animal Technology Co., Ltd and fed them under specific pathogen-free conditions at the Laboratory Animal Center of Sun Yat-sen University. SCCVII cells were used for tumor inoculation when they reached the exponential growth phase. For tumor models, 100 μL of FBS-free DMEM/F12 containing 5×10^5^ cells were injected into the shaved right flanks of C3H mice subcutaneously. When the tumor volume reached 50 mm^3^, tumor models were treated with PBS (untreated group) or antimouse-PD-1 (treated group)(Selleck,Houston,USA, clone:RMP1-14). These treatments were administered on days 7, 9, and 11 with the same dose, 200 μg/mouse i.p. All animal experiments were conducted in strict accordance with protocols approved by the Institutional Animal Care and Use Committee of Sun Yat-sen University (SYSU-IACUC-2025-002999).

### 5.10 *In Vivo* anti-IL-17 treatment and tissue collection

On day 12 post-tumor inoculation, mice bearing established tumors were randomized into treatment groups. The treatment group received an antimouse-IL-17A neutralizing antibody (Selleck,Houston,USA, clone:17F3, dose: 100 μg/mouse), while the control group received a corresponding isotype control antibody (Selleck,Houston,USA, clone:MOPC-21, dose: 100 μg/mouse). The treatment regimen consisted of three intraperitoneal (i.p.) injections administered at 3-day intervals.

At the study endpoint on day 33, saliva flow rate was measured. Mice were first anesthetized with an i.p. injection of sodium pentobarbital (50 mg/kg) and placed in a prone position. Salivation was then stimulated with pilocarpine hydrochloride (2 mg/kg). Following a 10-minute latency period, whole stimulated saliva was collected from the oral cavity for 10 minutes using pre-weighed cotton balls. Saliva volume was determined by the change in weight. At the designated experimental endpoints, mice were humanely euthanized. Specifically, euthanasia was induced by an intraperitoneal (i.p.) injection of an overdose of sodium pentobarbital (150 mg/kg), followed by cervical dislocation as a secondary physical method to ensure irreversible death. The submandibular glands were promptly harvested, with one lobe fixed in 4% paraformaldehyde for histological analysis and the other lobe snap-frozen in liquid nitrogen for molecular analysis. All animal carcasses were disposed of according to institutional biohazardous waste guidelines.

### 5.11 Flow cytometry analysis

For clinical samples, single-cell suspensions were prepared from fresh SG tissue. Briefly, tissues were mechanically minced and then enzymatically digested in a solution containing collagenase II (2 mg/mL; Worthington) and DNase I (4 mg/mL; Worthington) for 60 min at 37°C with continuous agitation. The resulting cell suspension was filtered through a 70-μm cell strainer to remove undigested tissue. Following centrifugation (400 ×*g*, 5 min), red blood cells were lysed by incubating the cell pellet with RBC Lysis Buffer for 1–2 min. The reaction was stopped with excess FACS buffer (PBS containing 2% FBS), and the cells were washed twice before being counted and resuspended for staining. For staining, cells were first incubated with Zombie Violet™ Fixable Viability Kit (BioLegend) for 15 min to enable discrimination of live and dead cells. After washing, non-specific antibody binding was blocked by incubating cells with a human Fc receptor blocking reagent (e.g., Human TruStain FcX™, BioLegend) for 20 min. Subsequently, cells were stained with a cocktail of the following fluorochrome-conjugated surface antibodies¹ for 30 min at 4°C in the dark: FITC anti-human CD45, Brilliant Violet 510™ anti-human CD3, Brilliant Violet 605™ anti-human CD19,Brilliant Violet 650™ anti-human CXCR3,PE anti-human CD25, PE-Cy7 anti-human CCR6, APC-Cy7 anti-human CD4, and Red Fluor 710 anti-human CD8. For intracellular staining of the transcription factor Foxp3, cells were fixed and permeabilized using a Foxp3/Transcription Factor Staining Buffer Set (eBioscience) according to the manufacturer’s protocol. Cells were then stained with PE-Cy7 anti-human Foxp3 (eBioscience).

For tumor-model mice, the bilateral submandibular glands of each mouse were separated, cut into small pieces, and digested in a solution containing collagenase II (2 mg/mL; Worthington) and DNase I (4 mg/mL; Worthington) for 60 min at 37°C with continuous agitation. A single-cell suspension was obtained by grinding tissue blocks, from which we selected live cells after incubation with Zombie dye at 4°C for 20 min. After centrifugation (400 ×*g*, 5 min), the cells were washed with buffer and blocked with antimouse CD16/32 at 4°C for 30 min. Subsequently, the cells were incubated with surface-staining fluorescent antibodies (PerCP anti-mouse CD45, PE-CY7 anti-mouse CD3, AF700 anti-mouse CD4, PE anti-mouse CD8a, APC anti-mouse CD19) at 4°C for 20 min.

Data were acquired on a BD LSRFortessa™ flow cytometer (BD Biosciences). Compensation and data analysis were performed using FlowJo™ software (v10, BD Life Sciences).

### 5.12 Statistical analysis

All quantitative data are presented as the mean ± standard error of the mean (SEM). Statistical analyses were performed with GraphPad Prism software (v.9.0, GraphPad Software, La Jolla, CA, USA). Comparisons between two independent groups were conducted using an unpaired two-tailed Student’s t-test. For comparisons among three or more groups, one-way analysis of variance (ANOVA) followed by an appropriate *post hoc* test (e.g., Tukey’s) was employed. To assess the relationship between two continuous variables between CD4^+^ cells,CD8^+^ cells and AQP5^+^ Cells, correlation analysis was performed. Pearson correlation coefficient (r) was calculated for normally distributed data, while Spearman rank correlation coefficient (ρ) was used for non-normally distributed data. A *P*-value less than 0.05 was considered statistically significant.

## Data Availability

The original contributions presented in the study are included in the article/**Supplementary Material**. Further inquiries can be directed to the corresponding authors.

## References

[1] Huo L Wang C Ding H Shi X Shan B Zhou R . Severe thyrotoxicosis induced by tislelizumab: A case report and literature review. Front Oncol. (2023) 13:1190491. doi: 10.3389/fonc.2023.1190491, PMID: 37849819 PMC10578961

[2] Zhou X Iwama S Kobayashi T Ando M Arima H . Risk of thyroid dysfunction in pd-1 blockade is stratified by the pattern of tgab and tpoab positivity at baseline. J Clin Endocrinol Metab. (2023) 108:e1056–e62. doi: 10.1210/clinem/dgad231, PMID: 37084392

[3] Iwamoto Y Kimura T Iwamoto H Sanada J Fushimi Y Katakura Y . Incidence of endocrine-related immune-related adverse events in Japanese subjects with various types of cancer. Front Endocrinol (Lausanne). (2023) 14:1079074. doi: 10.3389/fendo.2023.1079074, PMID: 36755909 PMC9899881

[4] Li Y Zang Y Fan T Li Z Li A Lv W . Transcriptomic signatures associated with autoimmune thyroiditis in papillary thyroid carcinoma and cancer immunotherapy-induced thyroid dysfunction. Comput Struct Biotechnol J. (2022) 20:2391–401. doi: 10.1016/j.csbj.2022.05.019, PMID: 35664236 PMC9125670

[5] Wu Z Shi M Shan F Li S Wang Y Xue K . Post-gastric cancer surgery hypophysitis and cortisol insufficiency after immunotherapy: A case series. Holistic Integr Oncol. (2026) 5:3. doi: 10.1007/s44178-025-00220-1, PMID: 41732346

[6] Zhou L Wei X . Ocular immune-related adverse events associated with immune checkpoint inhibitors in lung cancer. Front Immunol. (2021) 12:701951. doi: 10.3389/fimmu.2021.701951, PMID: 34504488 PMC8421677

[7] Gong Y Liu Y Jiang F Wang X . Ocular immune-related adverse events associated with pd-1 inhibitors: from molecular mechanisms to clinical management. Semin Ophthalmol. (2025) 40:288–305. doi: 10.1080/08820538.2024.2433636, PMID: 39606920

[8] Zhang Y Fang Y Wu J Huang G Bin J Liao Y . Pancreatic adverse events associated with immune checkpoint inhibitors: A large-scale pharmacovigilance analysis. Front Pharmacol. (2022) 13:817662. doi: 10.3389/fphar.2022.817662, PMID: 35431928 PMC9012537

[9] Xu Y Wen N Haddad RI Sonis ST Villa A . Comparisons of non-oral immune-related adverse events among patients with cancer with different oral toxicity profiles. Oncologist. (2024) 29:e382–e91. doi: 10.1093/oncolo/oyad279, PMID: 37874927 PMC10911904

[10] Mayer K Briese W Blieninger J Brossart P Bisht S Feldmann G . Development of skin rash predicts outcome of anti-pd-1- and anti-ctla4-based immune checkpoint inhibitor therapy in non-small cell lung cancer or squamous cell carcinoma of the head and neck: A single-center analysis. Oncol Res Treat. (2021) 44:538–46. doi: 10.1159/000518449, PMID: 34515189

[11] Hong AS Sarwar N Goldin RD Dhar A Possamai LA . Pembrolizumab-induced pancreatic exocrine insufficiency complicated by severe hepatic steatosis. Cureus. (2022) 14:e26596. doi: 10.7759/cureus.26596, PMID: 35936135 PMC9354921

[12] De Martin E Michot JM Papouin B Champiat S Mateus C Lambotte O . Characterization of liver injury induced by cancer immunotherapy using immune checkpoint inhibitors. J Hepatol. (2018) 68:1181–90. doi: 10.1016/j.jhep.2018.01.033, PMID: 29427729

[13] Cappelli LC Gutierrez AK Baer AN Albayda J Manno RL Haque U . Inflammatory arthritis and sicca syndrome induced by nivolumab and ipilimumab. Ann Rheum Dis. (2017) 76:43–50. doi: 10.1136/annrheumdis-2016-209595, PMID: 27307501 PMC5333990

[14] Holmberg KV Hoffman MP . Anatomy, biogenesis and regeneration of salivary glands. Monogr Oral Sci. (2014) 24:1–13. doi: 10.1159/000358776, PMID: 24862590 PMC4048853

[15] Pedersen AML Sørensen CE Proctor GB Carpenter GH Ekström J . Salivary secretion in health and disease. J Oral Rehabil. (2018) 45:730–46. doi: 10.1111/joor.12664, PMID: 29878444

[16] Warner BM Baer AN Lipson EJ Allen C Hinrichs C Rajan A . Sicca syndrome associated with immune checkpoint inhibitor therapy. Oncologist. (2019) 24:1259–69. doi: 10.1634/theoncologist.2018-0823, PMID: 30996010 PMC6738284

[17] Ortiz Brugués A Sibaud V Herbault-Barrés B Betrian S Korakis I De Bataille C . Sicca syndrome induced by immune checkpoint inhibitor therapy: optimal management still pending. Oncologist. (2020) 25:e391–e5. doi: 10.1634/theoncologist.2019-0467, PMID: 32043780 PMC7011671

[18] Higashi T Miyamoto H Yoshida R Furuta Y Nagaoka K Naoe H . Sjögren’s syndrome as an immune-related adverse event of nivolumab treatment for gastric cancer. Intern Med. (2020) 59:2499–504. doi: 10.2169/internalmedicine.4701-20, PMID: 32581160 PMC7662059

[19] Mavragani CP Moutsopoulos HM . Sicca syndrome following immune checkpoint inhibition. Clin Immunol. (2020) 217:108497. doi: 10.1016/j.clim.2020.108497, PMID: 32531346

[20] Caeyman A Vandekerckhove O Pat K Wynants J Weytjens K de Wergifosse I . Sjögren’s syndrome caused by pd-1 inhibition in a lung cancer patient. Case Rep Oncol. (2023) 16:1095–9. doi: 10.1159/000532098, PMID: 37900791 PMC10601759

[21] Le Burel S Champiat S Mateus C Marabelle A Michot JM Robert C . Prevalence of immune-related systemic adverse events in patients treated with anti-programmed cell death 1/anti-programmed cell death-ligand 1 agents: A single-centre pharmacovigilance database analysis. Eur J Cancer. (2017) 82:34–44. doi: 10.1016/j.ejca.2017.05.032, PMID: 28646772

[22] Bustillos H Indorf A Alwan L Thompson J Jung L . Xerostomia: an immunotherapy-related adverse effect in cancer patients. Support Care Cancer. (2022) 30:1681–7. doi: 10.1007/s00520-021-06535-9, PMID: 34562169

[23] Elad S Yarom N Zadik Y . Immunotherapy-related oral adverse effects: immediate sequelae, chronicity and secondary cancer. Cancers (Basel). (2023) 15:4781. doi: 10.3390/cancers15194781, PMID: 37835475 PMC10571987

[24] Nocturne G Mariette X . B cells in the pathogenesis of primary sjögren syndrome. Nat Rev Rheumatol. (2018) 14:133–45. doi: 10.1038/nrrheum.2018.1, PMID: 29416129

[25] Takahashi S Chieko X Sakai T Hirose S Nakamura M . Nivolumab-induced sialadenitis. Respirol Case Rep. (2018) 6:e00322. doi: 10.1002/rcr2.322, PMID: 29686875 PMC5899999

[26] Ghosn J Vicino A Michielin O Coukos G Kuntzer T Obeid M . A severe case of neuro-sjögren’s syndrome induced by pembrolizumab. J Immunother Cancer. (2018) 6:110. doi: 10.1186/s40425-018-0429-4, PMID: 30348223 PMC6196470

[27] Soliman A Hassan R Codreanu I Plaxe SC Dasanu CA . Sjogren’s syndrome due to immune checkpoint inhibitors (Icis): insights from a single-institution series and systematic review of the literature. J Oncol Pharm Pract. (2025) 31:1029–36. doi: 10.1177/10781552241271753, PMID: 39113536

[28] Le Burel S Champiat S Routier E Aspeslagh S Albiges L Szwebel TA . Onset of connective tissue disease following anti-pd1/pd-L1 cancer immunotherapy. Ann Rheum Dis. (2018) 77:468–70. doi: 10.1136/annrheumdis-2016-210820, PMID: 28242618

[29] Segawa T Motoshima T Yatsuda J Kurahashi R Fukushima Y Murakami Y . Sicca syndrome during ipilimumab and nivolumab therapy for metastatic renal cell carcinoma. IJU Case Rep. (2023) 6:147–9. doi: 10.1002/iju5.12573, PMID: 36874997 PMC9978085

[30] Buchholzer S Faure F Tcheremissinoff L Herrmann FR Lombardi T Ng SK . Novel multidisciplinary salivary gland society (Msgs) questionnaire: an international consensus. Laryngoscope. (2022) 132:322–31. doi: 10.1002/lary.29731, PMID: 34236085 PMC9291943

[31] Delporte C Bryla A Perret J . Aquaporins in salivary glands: from basic research to clinical applications. Int J Mol Sci. (2016) 17:166. doi: 10.3390/ijms17020166, PMID: 26828482 PMC4783900

[32] Ramos-Casals M Maria A Suárez-Almazor ME Lambotte O Fisher BA Hernández-Molina G . Sicca/sjögren’s syndrome triggered by pd-1/pd-L1 checkpoint inhibitors. Data from the international immunocancer registry (Icir). Clin Exp Rheumatol. (2019) 37 Suppl 118:114–22. 31464670

[33] Coulie PG Van den Eynde BJ van der Bruggen P Boon T . Tumour antigens recognized by T lymphocytes: at the core of cancer immunotherapy. Nat Rev Cancer. (2014) 14:135–46. doi: 10.1038/nrc3670, PMID: 24457417

[34] Pringle S van der Vegt B Wang X van Bakelen N Hiltermann TJN Spijkervet FKL . Lack of conventional acinar cells in parotid salivary gland of patient taking an anti-pd-L1 immune checkpoint inhibitor. Front Oncol. (2020) 10:420. doi: 10.3389/fonc.2020.00420, PMID: 32300556 PMC7142242

[35] Baldini C Fulvio G La Rocca G Ferro F . Update on the pathophysiology and treatment of primary sjögren syndrome. Nat Rev Rheumatol. (2024) 20:473–91. doi: 10.1038/s41584-024-01135-3, PMID: 38982205

[36] Fragoulis GE Zampeli E Moutsopoulos HM . Igg4-related sialadenitis and sjögren’s syndrome. Oral Dis. (2017) 23:152–6. doi: 10.1111/odi.12526, PMID: 27318181

[37] Ibrahem HM . T cell roles and activity in chronic sclerosing sialadenitis as igg4-related disease: current concepts in immunopathogenesis. Autoimmune Dis. (2022) 2022:5689883. doi: 10.1155/2022/5689883, PMID: 35769404 PMC9236833

[38] Borys SM Reilly SP Magill I Zemmour D Brossay L . Nk cells restrain cytotoxic cd8(+) T cells in the submandibular gland via pd-1-pd-L1. Sci Immunol. (2024) 9:eadl2967. doi: 10.1126/sciimmunol.adl2967, PMID: 39705335 PMC12099074

[39] Ju M Zhang J Deng Z Wei M Ma L Chen T . Prophylactic il-23 blockade uncouples efficacy and toxicity in dual ctla-4 and pd-1 immunotherapy. J Immunother Cancer. (2024) 12:e009345. doi: 10.1136/jitc-2024-009345, PMID: 39089739 PMC11293404

[40] Lozano AX Chaudhuri AA Nene A Bacchiocchi A Earland N Vesely MD . T cell characteristics associated with toxicity to immune checkpoint blockade in patients with melanoma. Nat Med. (2022) 28:353–62. doi: 10.1038/s41591-021-01623-z, PMID: 35027754 PMC8866214

[41] Fu S Guo Z Xu X Li Y Choi S Zhao P . Protective effect of low-intensity pulsed ultrasound on immune checkpoint inhibitor-related myocarditis via fine-tuning cd4(+) T-cell differentiation. Cancer Immunol Immunother. (2024) 73:15. doi: 10.1007/s00262-023-03590-5, PMID: 38236243 PMC10796578

[42] Hu X Bukhari SM Tymm C Adam K Lerrer S Henick BS . Inhibition of il-25/il-17ra improves immune-related adverse events of checkpoint inhibitors and reveals antitumor activity. J Immunother Cancer. (2024) 12:e008482. doi: 10.1136/jitc-2023-008482, PMID: 38519059 PMC10961528

[43] Tsukamoto H Komohara Y Tomita Y Miura Y Motoshima T Imamura K . Aging-associated and cd4 T-cell-dependent ectopic cxcl13 activation predisposes to anti-pd-1 therapy-induced adverse events. Proc Natl Acad Sci U.S.A. (2022) 119:e2205378119. doi: 10.1073/pnas.2205378119, PMID: 35858347 PMC9303859

[44] Chuah S Lee J Song Y Kim HD Wasser M Kaya NA . Uncoupling immune trajectories of response and adverse events from anti-pd-1 immunotherapy in hepatocellular carcinoma. J Hepatol. (2022) 77:683–94. doi: 10.1016/j.jhep.2022.03.039, PMID: 35430299

[45] Mima Y Ohtsuka T Ebato I Nakazato Y Norimatsu Y . A case of bullous pemphigoid with significant infiltration of cd4-positive T cells during treatment with pembrolizumab, accompanied by pembrolizumab-induced multi-organ dysfunction. Diagnostics (Basel). (2024) 14:1958. doi: 10.3390/diagnostics14171958, PMID: 39272742 PMC11394162

[46] Yue M Li C Li G . New advances in the study of pd-1/pd-L1 inhibitors-induced liver injury. Int Immunopharmacol. (2024) 131:111799. doi: 10.1016/j.intimp.2024.111799, PMID: 38460297

[47] Zhao Y Wucherpfennig KW . Tissue-resident T cells in clinical response and immune-related adverse events of immune checkpoint blockade. Clin Cancer Res. (2024) 30:5527–34. doi: 10.1158/1078-0432.Ccr-23-3296, PMID: 39404858 PMC11649445

[48] Kim ST Chu Y Misoi M Suarez-Almazor ME Tayar JH Lu H . Distinct molecular and immune hallmarks of inflammatory arthritis induced by immune checkpoint inhibitors for cancer therapy. Nat Commun. (2022) 13:1970. doi: 10.1038/s41467-022-29539-3, PMID: 35413951 PMC9005525

[49] Xin Z You L Na F Li J Chen M Song J . Immunogenetic variations predict immune-related adverse events for pd-1/pd-L1 inhibitors. Eur J Cancer. (2023) 184:124–36. doi: 10.1016/j.ejca.2023.01.034, PMID: 36917924

[50] Abed A Law N Calapre L Lo J Bhat V Bowyer S . Human leucocyte antigen genotype association with the development of immune-related adverse events in patients with non-small cell lung cancer treated with single agent immunotherapy. Eur J Cancer. (2022) 172:98–106. doi: 10.1016/j.ejca.2022.05.021, PMID: 35759816

[51] Jin Y Chen DL Wang F Yang CP Chen XX You JQ . The predicting role of circulating tumor DNA landscape in gastric cancer patients treated with immune checkpoint inhibitors. Mol Cancer. (2020) 19:154. doi: 10.1186/s12943-020-01274-7, PMID: 33126883 PMC7596978

[52] Pardoll D . Releasing the brakes on antitumor immune response. Science. (1996) 271:1691. doi: 10.1126/science.271.5256.1691, PMID: 8596929

[53] Reschke R Gussek P Boldt A Sack U Köhl U Lordick F . Distinct immune signatures indicative of treatment response and immune-related adverse events in melanoma patients under immune checkpoint inhibitor therapy. Int J Mol Sci. (2021) 22:8017. doi: 10.3390/ijms22158017, PMID: 34360781 PMC8348898

[54] Okiyama N Tanaka R . Immune-related adverse events in various organs caused by immune checkpoint inhibitors. Allergol Int. (2022) 71:169–78. doi: 10.1016/j.alit.2022.01.001, PMID: 35101349

[55] Baumjohann D Brossart P . T follicular helper cells: linking cancer immunotherapy and immune-related adverse events. J Immunother Cancer. (2021) 9:e002588. doi: 10.1136/jitc-2021-002588, PMID: 34112740 PMC8194326

[56] Reschke R Shapiro JW Yu J Rouhani SJ Olson DJ Zha Y . Checkpoint blockade-induced dermatitis and colitis are dominated by tissue-resident memory T cells and th1/tc1 cytokines. Cancer Immunol Res. (2022) 10:1167–74. doi: 10.1158/2326-6066.Cir-22-0362, PMID: 35977003 PMC9530647

[57] Llewellyn HP Arat S Gao J Wen J Xia S Kalabat D . T cells and monocyte-derived myeloid cells mediate immunotherapy-related hepatitis in a mouse model. J Hepatol. (2021) 75:1083–95. doi: 10.1016/j.jhep.2021.06.037, PMID: 34242700

[58] Kim KH Hur JY Cho J Ku BM Koh J Koh JY . Immune-Related Adverse Events Are Clustered into Distinct Subtypes by T-Cell Profiling before and Early after Anti-Pd-1 Treatment. Oncoimmunology. (2020) 9:1722023. doi: 10.1080/2162402x.2020.1722023, PMID: 32076579 PMC6999841

[59] Dulos J Carven GJ van Boxtel SJ Evers S Driessen-Engels LJ Hobo W . Pd-1 blockade augments th1 and th17 and suppresses th2 responses in peripheral blood from patients with prostate and advanced melanoma cancer. J Immunother. (2012) 35:169–78. doi: 10.1097/CJI.0b013e318247a4e7, PMID: 22306905

[60] Dimitriou F Cheng PF Saltari A Schaper-Gerhardt K Staeger R Haunerdinger V . A targetable type iii immune response with increase of il-17a expressing cd4(+) T Cells is associated with immunotherapy-induced toxicity in melanoma. Nat Cancer. (2024) 5:1390–408. doi: 10.1038/s43018-024-00810-4, PMID: 39210005 PMC11424476

[61] Anvar MT Rashidan K Arsam N Rasouli-Saravani A Yadegari H Ahmadi A . Th17 cell function in cancers: immunosuppressive agents or anti-tumor allies? Cancer Cell Int. (2024) 24:355. doi: 10.1186/s12935-024-03525-9, PMID: 39465401 PMC11514949

[62] McCoy SS Giri J Das R Paul PK Pennati A Parker M . Minor salivary gland mesenchymal stromal cells derived from patients with sjögren’s syndrome deploy intact immune plasticity. Cytotherapy. (2021) 23:301–10. doi: 10.1016/j.jcyt.2020.09.008, PMID: 33262072 PMC8728747

[63] Zhao L Jin S Wang S Zhang Z Wang X Chen Z . Tertiary lymphoid structures in diseases: immune mechanisms and therapeutic advances. Signal Transduct Target Ther. (2024) 9:225. doi: 10.1038/s41392-024-01947-5, PMID: 39198425 PMC11358547

[64] Sato Y Silina K van den Broek M Hirahara K Yanagita M . The roles of tertiary lymphoid structures in chronic diseases. Nat Rev Nephrol. (2023) 19:525–37. doi: 10.1038/s41581-023-00706-z, PMID: 37046081 PMC10092939

[65] Dong Y Wang T Wu H . Tertiary lymphoid structures in autoimmune diseases. Front Immunol. (2023) 14:1322035. doi: 10.3389/fimmu.2023.1322035, PMID: 38259436 PMC10800951

[66] Astorri E Scrivo R Bombardieri M Picarelli G Pecorella I Porzia A . Cx3cl1 and cx3cr1 expression in tertiary lymphoid structures in salivary gland infiltrates: fractalkine contribution to lymphoid neogenesis in sjogren’s syndrome. Rheumatol (Oxford). (2014) 53:611–20. doi: 10.1093/rheumatology/ket401, PMID: 24324211

[67] Loureiro-Amigo J Palacio-García C Martínez-Gallo M Martínez-Valle F Ramentol-Sintas M Soláns-Laqué R . Utility of lymphocyte phenotype profile to differentiate primary sjögren’s syndrome from sicca syndrome. Rheumatol (Oxford). (2021) 60:5647–58. doi: 10.1093/rheumatology/keab170, PMID: 33620072

[68] Liu X Wu W Fang L Liu Y Chen W . Tnf-A; Inhibitors and other biologic agents for the treatment of immune checkpoint inhibitor-induced myocarditis. Front Immunol. (2022) 13:922782. doi: 10.3389/fimmu.2022.922782, PMID: 35844550 PMC9283712

[69] Fa’ak F Buni M Falohun A Lu H Song J Johnson DH . Selective immune suppression using interleukin-6 receptor inhibitors for management of immune-related adverse events. J Immunother Cancer. (2023) 11:e006814. doi: 10.1136/jitc-2023-006814, PMID: 37328287 PMC10277540

[70] Nayar S Turner JD Asam S Fennell E Pugh M ColaFrancesco S . Molecular and spatial analysis of tertiary lymphoid structures in sjogren’s syndrome. Nat Commun. (2025) 16:5. doi: 10.1038/s41467-024-54686-0, PMID: 39747819 PMC11697438

[71] Ma D Feng Y Lin X . Immune and non-immune mediators in the fibrosis pathogenesis of salivary gland in sjögren’s syndrome. Front Immunol. (2024) 15:1421436. doi: 10.3389/fimmu.2024.1421436, PMID: 39469708 PMC11513355

[72] Peng B Wang L Pan S Kang J Wei L Li B . Metformin attenuates partial epithelial-mesenchymal transition in salivary gland inflammation via pi3k/akt/gsk3β/snail signaling axis. Inflammation. (2025) 48:1525–37. doi: 10.1007/s10753-024-02142-y, PMID: 39269669

[73] Sarrand J Soyfoo MS . Involvement of epithelial-mesenchymal transition (Emt) in autoimmune diseases. Int J Mol Sci. (2023) 24:14481. doi: 10.3390/ijms241914481, PMID: 37833928 PMC10572663

[74] Wang L Zhong NN Wang X Peng B Chen Z Wei L . Metformin attenuates tgf-B1-induced fibrosis in salivary gland: A preliminary study. Int J Mol Sci. (2023) 24:16260. doi: 10.3390/ijms242216260, PMID: 38003450 PMC10671059

[75] Altrieth AL O’Keefe KJ Gellatly VA Tavarez JR Feminella SM Moskwa NL . Identifying fibrogenic cells following salivary gland obstructive injury. bioRxiv. (2023) 11:1190386. doi: 10.1101/2023.03.09.531751, PMID: 37287453 PMC10242138

[76] Sisto M Lorusso L Tamma R Ingravallo G Ribatti D Lisi S . Interleukin-17 and -22 synergy linking inflammation and emt-dependent fibrosis in sjögren’s syndrome. Clin Exp Immunol. (2019) 198:261–72. doi: 10.1111/cei.13337, PMID: 31165469 PMC6797899

[77] Sisto M Lorusso L Ingravallo G Ribatti D Lisi S . Tgfβ1-smad canonical and -erk noncanonical pathways participate in interleukin-17-induced epithelial-mesenchymal transition in sjögren’s syndrome. Lab Invest. (2020) 100:824–36. doi: 10.1038/s41374-020-0373-z, PMID: 31925325

[78] Muñoz Forti K Weisman GA Jasmer KJ . Cell type-specific transforming growth factor-B (Tgf-B) signaling in the regulation of salivary gland fibrosis and regeneration. J Oral Biol Craniofac Res. (2024) 14:257–72. doi: 10.1016/j.jobcr.2024.03.005, PMID: 38559587 PMC10979288

[79] Zhou J Felix FA Jiang Y Li D Kim MC Jang D . Altered characteristics of regulatory T cells in target tissues of sjögren’s syndrome in murine models. Mol Immunol. (2024) 174:47–56. doi: 10.1016/j.molimm.2024.08.003, PMID: 39197397 PMC11500054

[80] Langthasa J Guan L Jinagal SL Le QT . Salivary gland stem/progenitor cells: advancing from basic science to clinical applications. Cell Regener. (2025) 14:4. doi: 10.1186/s13619-025-00221-5, PMID: 39856475 PMC11759724

[81] Huang L Zhang Y Kuang F Xi Q Chen Y Xu W . Cp-25 ameliorates dysfunction in the M(3)R-ip(3)R-ca(2+)-aqp5 signaling pathway by inhibiting ers in primary sjögren’s syndrome. Int Immunopharmacol. (2026) 168:115804. doi: 10.1016/j.intimp.2025.115804, PMID: 41207101

[82] Barrera MJ Aguilera S Castro I González S Carvajal P Molina C . Endoplasmic reticulum stress in autoimmune diseases: can altered protein quality control and/or unfolded protein response contribute to autoimmunity? A critical review on sjögren’s syndrome. Autoimmun Rev. (2018) 17:796–808. doi: 10.1016/j.autrev.2018.02.009, PMID: 29890347

[83] Chibly AM Aure MH Patel VN Hoffman MP . Salivary gland function, development, and regeneration. Physiol Rev. (2022) 102:1495–552. doi: 10.1152/physrev.00015.2021, PMID: 35343828 PMC9126227

[84] Qu XM Wu ZF Pang BX Jin LY Qin LZ Wang SL . From nitrate to nitric oxide: the role of salivary glands and oral bacteria. J Dent Res. (2016) 95:1452–6. doi: 10.1177/0022034516673019, PMID: 27872324

[85] Monsour EP Pothen J Balaraman R . A novel approach to the treatment of pembrolizumab-induced psoriasis exacerbation: A case report. Cureus. (2019) 11:e5824. doi: 10.7759/cureus.5824, PMID: 31754559 PMC6827694

[86] Liu C Liu R Wang B Lian J Yao Y Sun H . Blocking IL-17a enhances tumor response to anti-pd-1 immunotherapy in microsatellite stable colorectal cancer. J Immunother Cancer. (2021) 9:e001895. doi: 10.1136/jitc-2020-001895, PMID: 33462141 PMC7813395

[87] Tanaka R Ichimura Y Kubota N Saito A Nakamura Y Ishitsuka Y . Activation of cd8 T cells accelerates anti-pd-1 antibody-induced psoriasis-like dermatitis through IL-6. Commun Biol. (2020) 3:571. doi: 10.1038/s42003-020-01308-2, PMID: 33060784 PMC7567105

