# Supplementary material for: Immunotherapy-induced sialadenitis: sjögren’s syndrome or a new sialadenitis
Source: Front Immunol. 2026 Feb 27;17:1755419. doi: 10.3389/fimmu.2026.1755419 (PMC12982336; doi:10.3389/fimmu.2026.1755419)
Supplement: Supplementary file 1 [file Supplementaryfile1.pdf]

Supplementary material

Supplementary Figure 1.

A

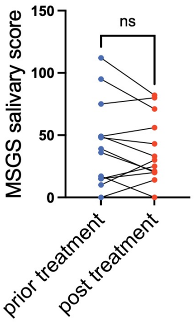

Supplementary Figure 2.

A Panel 1

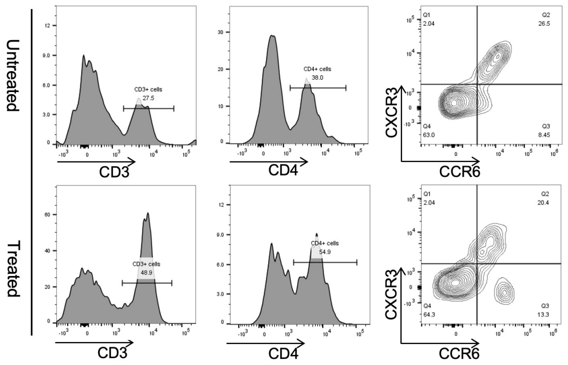

B Panel 2

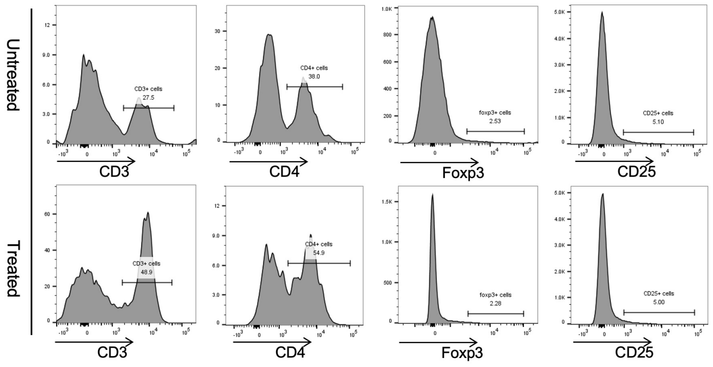

C

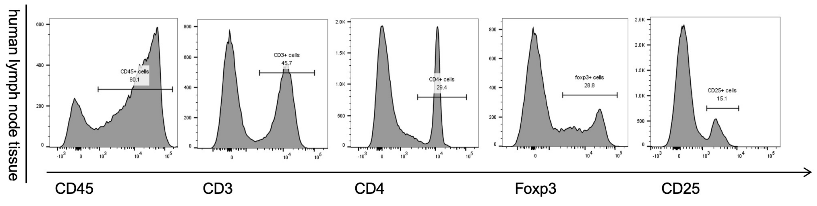

Supplementary Figure 3.

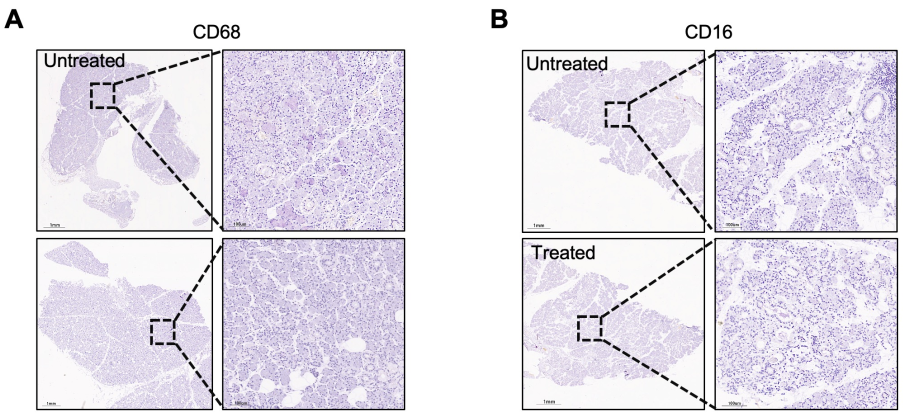

Supplementary Figure 1. Assessment of salivary gland function in patients prior- and post- ICIs. (A) Salivary gland function was evaluated using the Michigan Salivary Gland Scale(MSGS) questionnaire at prior-treatment and post-treatment. The scores were compared to analyze the changes. Data represent mean ± SEM.

Supplementary Figure 2. (A)Representative immunohistochemical (IHC) staining for CD68(macrophages marker), CD16 (neutrophils marker) in salivary gland tissues. Positive staining is indicated by brown diaminobenzidine (DAB), and sections were counterstained with hematoxylin (blue).Scale bars, 1 mm (1×) and 100 μm (20×).

Supplementary Figure 3. (A)(B) Representative flow cytometry plots and histograms illustrating the sequential gating hierarchy for Panel 1 (Th subsets) and Panel 2 (Tregs). (C) Positive control validation. Representative histograms of human lymph node tissue stained with Panel 2 demonstrate distinct positive populations for CD45,CD3,CD4,CD25 and FOXP3.
